# Integrative Analysis of Metabolomics and Transcriptomics Data: A Unified Model Framework to Identify Underlying System Pathways

**DOI:** 10.1371/journal.pone.0072116

**Published:** 2013-09-25

**Authors:** Kasper Brink-Jensen, Søren Bak, Kirsten Jørgensen, Claus Thorn Ekstrøm

**Affiliations:** 1 Department of Mathematical Sciences, University of Copenhagen, Copenhagen, Denmark; 2 Department of Plant Biology and Biotechnology, University of Copenhagen, Copenhagen, Denmark; 3 Department of Biostatistics, University of Southern Denmark, Odense, Denmark; University of Manchester, United Kingdom

## Abstract

The abundance of high-dimensional measurements in the form of gene expression and mass spectroscopy calls for models to elucidate the underlying biological system. For widely studied organisms like yeast, it is possible to incorporate prior knowledge from a variety of databases, an approach used in several recent studies. However if such information is not available for a particular organism these methods fall short. In this paper we propose a statistical method that is applicable to a dataset consisting of Liquid Chromatography-Mass Spectroscopy (LC-MS) and gene expression (DNA microarray) measurements from the same samples, to identify genes controlling the production of metabolites. Due to the high dimensionality of both LC-MS and DNA microarray data, dimension reduction and variable selection are key elements of the analysis. Our proposed approach starts by identifying the basis functions (“building blocks”) that constitute the output from a mass spectrometry experiment. Subsequently, the weights of these basis functions are related to the observations from the corresponding gene expression data in order to identify which genes are associated with specific patterns seen in the metabolite data. The modeling framework is extremely flexible as well as computationally fast and can accommodate treatment effects and other variables related to the experimental design. We demonstrate that within the proposed framework, genes regulating the production of specific metabolites can be identified correctly unless the variation in the noise is more than twice that of the signal.

## Introduction

Metabolites are the products of cell metabolism and their functions are highly diverse. The profile of metabolites shows the current physiological state of a cell and is the end result of the upstream biological information that flows from the biological processes going from the genome over the transcriptome and proteome to the metabolome.

We wish to combine data from transcriptomics and metabolomics into one experimental setup in order to generate hypotheses about the regulatory processes between different molecular levels. While the biological processes between different levels of “omics” are highly complex, a combined analysis of metabolite and gene expression data will help discover and elucidate the underlying regulatory networks and identify genes that influence the metabolome because they – directly or indirectly – are involved in the metabolism.

Gene expression studies measure the simultaneous expression of up to thousands of genes and can be used to identify which genes are up- or down-regulated under certain conditions. Metabolomic studies provide information on the metabolites found within a biological sample – for example from mass spectrometry data – and can be used to discriminate between the amount and types of metabolites in different samples or under different conditions. These two “omics”-approaches address questions at different biological levels, but they both seek to uncover the underlying systems biology and when combined they can be succesful in predicting gene functions or identifying gene-metabolite associations [1], [2].

The idea of coupling data from different aspects of the same biological system – a term known as integrative analysis or “multi-omics” – is not new and several recent publications apply this idea to identify gene function or gene-to-metabolite networks for for example plant and cancer cells [2]–[5]. The integrated data analysis methods all need to reduce the dimensionality of the data (either of each type of data separately or by combining the two data types into a single normalized “dimensionless” dataset prior to dimension reduction) before multivariate correlation analysis, clustering methods, or for example self-organizing maps are used to identify groups of associated genes and metabolites [4], [6], [7]. Other approaches use prior knowledge (*e.g.*, previously identified regulatory pathways or previously hypothesized gene-metabolite relationship) to validate findings [6], [8]. The recent tool, MassTRIX, also uses existing annotation information from KEGG (Kyoto Encyclopedia of Genes and Genomes) to visualize combined transcriptome and metabolome data [9].

The references mentioned above typically uses some kind of correlation measure to describe the association between the two types of data, but multivariate methods like partial least squares (PLS) regression have also been used for integrated data analysis [10]. In PLS, combinations of explanatory variables are computed and these combinations are subsequently used as predictors in a statistical model. However, PLS is not without caveats since the main focus is on prediction and not on identifying the underlying system and interpreting the effect of individual genes and the underlying biological system may be difficult at best. When PLS is applied to full functional mass spectrometry data it results in components that are a mix of different mass-time combinations so it is impossible to infer how a set of genes influence a particular chemical compound and the underlying basis functions often lack a meaningful interpretation compared to the original data. A variation of these methods, co-inertia analysis (CIA) is based on maximizing covariance between PCA (principal component analysis) components of two separate datasets, which again could make interpretation difficult [11]. Common for all these methods is that they try to discover relationships between metabolomics and transcriptomics data without describing the underlying biological system except for the fact that the metabolites are the end product and hence are possibly influenced by one or more genes working on a level upstream from the metabolites.

In this paper, we will present a statistical modeling approach to model the association between gene expression and metabolite development as measured by DNA microarrays and Liquid Chromatography-Mass Spectroscopy (LC-MS), respectively. The idea is to use matrix decomposition to identify metabolite “building block” that constitute the observed metabolite profile data and then combine these results with a multivariate linear model with the gene expression data as possible predictors (properly regularized to prevent overfitting). This combined approach has the nice properties that we are 1) able to identify which metabolites occur in unison between different experimental setups, 2) we are able to handle complex experimental designs through the statistical model, 3) we get information about which genes are associated with each metabolite as well as the individual magnitude of their association, and 4) we do not require information from existing databases on known pathways or annotations. Thus, our proposed modeling approach can be used not only to validate previously known gene-to-metabolite networks but also to uncover new networks for organisms that are less investigated than, say, yeast or *Arabidopsis* and to describe the strength of these relationship. In addition, the underlying biological assumption that the metabolites are the end product of a complex biological process is kept in mind because the two datatypes are not assimilated into a single dimensionless dataset. Instead, the functional relationship where the gene expression levels can influence the metabolites forms the basis of the underlying model.

The paper is structured as follows: in the methods section we describe how a metabolite matrix decomposition can be combined with a regularization technique to model the associations between gene expression and metabolite profile data. The simulation section describes a simulation setup to show how effective our method is in identifying the correct relationships between genes and metabolites for various signal-to-noise ratios. In applications we apply the method to a Cassava dataset and the results section presents the results from the simulations and application. In the discussion we discuss advantages and disadvantages of the proposed method. Example code for running the analyses in R and MATLAB can be found at www.biostatistics.dk/ida.

## Results

**Figure 1:**
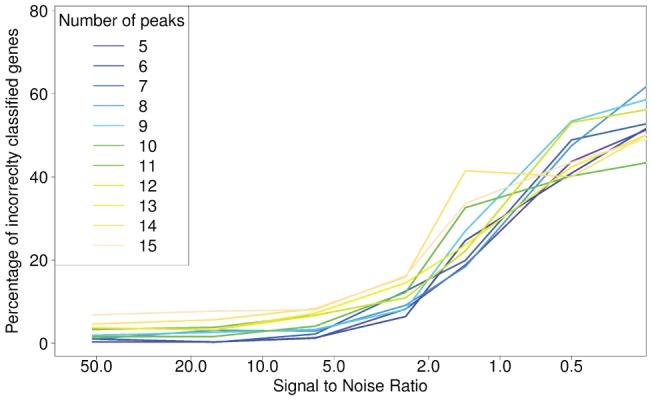
Results of simulation study. Each line represents the error rate for various signal-to-noise ratios for a given number of peaks in the simulated spectra. Each combination of peaks and noise ratio was run 300 times. 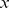 axis on log scale to help visualization.

To demonstrate the validity of the proposed method a simulation study was undertaken. Our primary focus is whether the approach is able to identify the correct pathways that govern the underlying relationships among gene expression levels and peaks in the simulated spectra. We also performed two other simulations to examine the robustness of the method. In one scenario we increased the number of genes, while the other investigated how the method performs if no associations between genes and peaks in the spectra are present. Each combination of 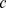 peaks and 7 noise levels was run 300 times and the results are shown in Figure 1. A decomposition with “number-of-peaks +1” components was used and the resulting mixing matrix was subsequently used as response in “number-of-peaks” models with the expression from 1000 simulated genes as predictors. The first selected gene from each model was compared with the gene known to be associated with the component, and if it did not match perfectly it was scored as an error. The percentage of the 300 simulations that resulted in errors is our error/misclassification rate. Each line in the figure represents a setup with a given number of peaks and the percentage of incorrectly classified genes are shown for shown for various signal-to-noise ratios (SNR) ranging form 52 to 0.1. Note that the 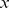-axis in Figure 1 is on a log scale to better differentiate the results for both low SNRs and high SNRs.

The percentage of correctly classified genes was very high, more than 95% on average when the SNR was higher than 5. With a SNR of 52 the correct genes were identified in 97.6% on average of all cases, ranging from 99.7% with 5 peaks to 93.2% with 15 peaks, decreasing slightly to 97.1% and 95.3% for SNR 16 and 6, respectively. From SNR 2.5 the percentage of correctly selected genes decreased more rapidly from 88.6% to 74.1% SNR 1.4, 53.9% SNR 0.5 and 38.9% for SNR 0.1.

Increasing the number of peaks generally resulted in larger error rates while the effect of noise appears independent of the number of peaks. This was most notable with high SNR. For lower SNR, this trend became less clear, in fact with SNR 0.5 the order of the lines corresponding to different number of peaks changed order: the error was 43.7% with 5 peaks and 43.5% with 15. The effect of lower SNR can be seen in Figure 1, from SNR 52 to 6 mean errors are below 5% with range about 7%. From SNR 2.5 to 0.1 the effect of noise appears non-linear, and error range increases to 31.8%.

**Figure 2:**
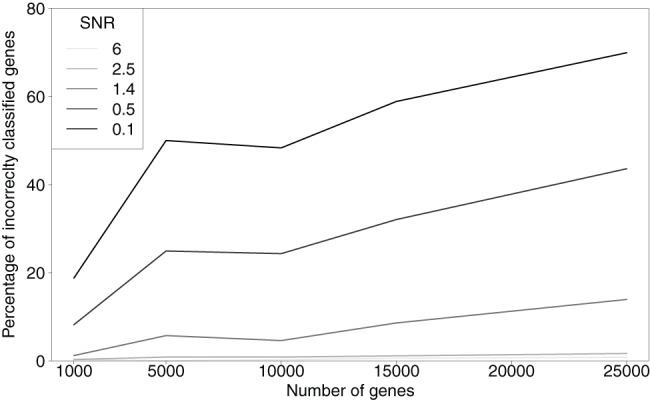
Results of simulation study. All simulations used 5 peaks and was run 300 times for each combination of noise and genes.

The effect of increasing the number of genes from 1000 to 25000 can be seen in Figure 2. More genes does decrease the precision of the model in particular when the number of genes is low. However, as soon as the initial number of genes is large (5000) then the noise in the data is far more influential on the error rate than the number of genes. With SNR 6 the error went from 0.3% using 5000 genes to 0.8% with 25000 genes. With lower SNR the effect of more genes affected the performance to a higher degree, with SNR 0.5 the error ranged from 8.2% to 43.6%. The effect of increasing the number of genes from 1000 to 25000 can be seen in Figure 2. More genes does only decrease the precision of the model slightly, however the noise in the data is substantially more influential. With SNR 6 the error went from 0.3% using 1000 genes to 0.8% with 25000 genes. With lower SNR the effect of more genes affected the performance to a higher degree, with SNR 0.5 the error ranged from 8.2% to 43.6%.

Finally we wanted to investigate the performance of the model when no associations between genes and peaks in the spectra are present. We did this by generating data as previously described with 10000 genes, a SNR of 20 and 5 peaks. After generating the spectra we removed the 5 influential genes, and used a random subset of 1000 genes from the remaining 9995. Following gene selection by LARS we fitted a multiple regression model with the selected genes in order to obtain a 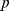-value for the gene selected first by LARS. This procedure was run 300000 times. Out of the possible 9995 genes, only 475 genes were among the first selected genes with a frequency ranging from 1 to 30199 times (mean 3158 and standard deviation 6365). Few genes (87) were selected more than 5000 times and only 5 of 9995 were selected approximately 30000 times. The average 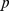-values from the multiple regression model was 0.49 with standard deviation 0.11. P-values centered closely around 0.5 and few genes selected very often indicates that the method is robust against unfortunate subset compositions. Shrinkage methods do not produce 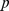-values and therefore the strength is not known. The genes selected are the best ones even if selected from a set with no associations to the response. However this result suggests that variable importance can be employed to quantify the significance of the found association.

**Figure 3:**
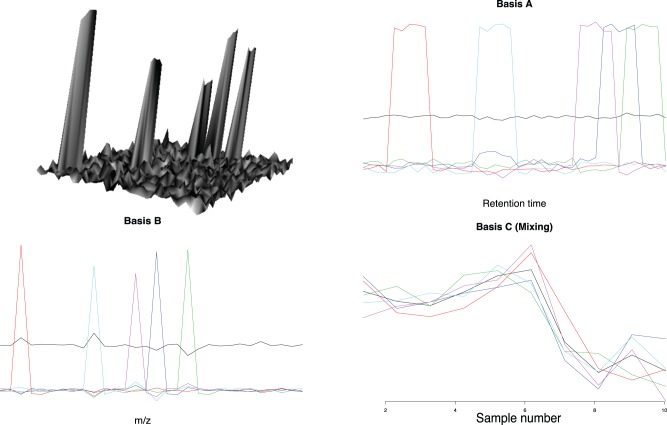
Example of simulated spectra with 5 peaks. Each peak height is controlled by a the expression level of a specific gene. Background is random normal noise. The simulation study used 10 replicates to construct the 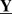 tensor, one is shown in the upper left panel. PARAFAC decomposition of the simulated spectra, a 6 component (5 for the peaks and 1 for the noise) model was used. In a real-world application basis 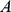 and 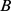, would be used to identify the compounds in the sample based on their combination of retention time and 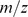 value. Basis 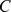 is used as response in a regression model with gene expressions as predictors, and for each sample unit a relative weight of the of the components can be seen.

As mentioned previously the results from the analysis are directly interpretable with respect to the original data. Assume, for instance, that one sample like the the upper left panel in Figure 3 corresponds to one biological sample out of several. At the end of the analysis we know how the individual components/features look, their positions as described by retention time and 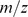 makes it possible to identify the metabolic compounds that represent the peaks in the LC-MS spectra. For example, the red colored basis function in Basis A and B can easily be identified in the original spectra as the peak furthest to the left. At the same time we know the weights of all the basis functions required to reconstruct the original samples. Using the corresponding mixing component (also in red) we are able to find genes associated with that specific peak.

## Discussion

The underlying assumption in our setup is that the gene expression levels may influence the downstream production of metabolites. We have clearly demonstrated that within this framework, genes regulating the production of specific metabolites can be identified even though the proposed method uses very few assumptions. In that sense, our approach resembles unsupervised learning techniques since we both extract possible basis functions from the spectra data and identify associations between the weights of the basis functions and the gene expression levels.

In the simulation study we achieved very high accuracy in identifying the genes known to control the peak heights (metabolite content) in the generated spectra. As expected, the accuracy was was highest when the signal-to-noise ratio was high and the error rate was less than 20% until a ratio around 2. Even when the signal-to-noise ratio is less than one the error rate generally is less than 60%.

Not surprisingly, the performance of the model decreases when more peaks are present (see Figure 1). This is a result of poorer separation by parallel factor analysis (PARAFAC), which is to be expected as partial or even full peak overlaps become more frequent. Note that explained variation can still be high even if separation of the tensor into the original components is poor, in the sense that the estimated components do not resemble the original components. This can occur if the estimated components are mixtures of true components or mixtures of noise and true components. The simulated spectra were notably smaller (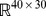) than most real-world spectra purely to reduce computing time for the simulations. In practice the peaks will be distributed over a larger surface, resulting in greater separation performance in most experiments.

For high SNRs there was a clear trend of larger error rate with more peaks. This trend is obscured when SNR reaches approximately 1, *i.e.* when the variation in signal and noise is of the same magnitude. At this point the performance of the PARAFAC separation becomes less influential than the noise added to the gene expression before modeling.

The variation of the model precision must increase with the magnitude of the noise added to the simulated gene expression data. However, the impact of this will be low since gene expression data from real experiments can to some extent be cleaned of noise and often exhibit relatively low noise compared to the values tried in our simulations [12].

The number of components for the PARAFAC model, 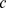, should be carefully selected to ensure that the mixing matrix does indeed represent the mixing of individual chemical compounds. With simulated data this is trivial as the true number of components is known. With real data this can be a daunting task if the peaks in the spectra are very dense. Too many components will mean that more than one component will describe a single feature, while too few will result in components that describe more than one feature.

It is worth noting that a PARAFAC model with “sufficient” components might return basis functions that capture more than one feature. In this case the genes selected by the analysis are assumed to influence all features in the original spectra. We argue that it is not unreasonable to assume that a useful separation can be achieved, and in any setting, decompositions of this type require a qualified estimate of the number of components. A first step is likely to be a visual inspection of the spectra followed by fitting a few models with different (but not much) components. Optimization of the number of factors has received some research interest, [13] and [14] both propose algorithms to aid in the selection. In extreme cases it might be worthwhile to divide the original tensor of (aligned) spectra into a number of sub-tensors and apply the proposed model to each of these. Preprocessing such as alignment and baseline correction can also aid in reducing the error.

In this paper, we have used PARAFAC to decompose the metabolomics data and least angle regression (LARS) to reduce the dimension of the gene expression data. However, essentially we have formulated a framework which accommodates different methods for both the decomposition and the covariate selection. Any method that can decompose the metabolite data could in principle be used for the matrix decomposition and any regularization method could be used for the covariate selection. This leaves the investigator with a large and flexible toolbox where the choice of method should be based on which qualities of the methods that are relevant for the situation at hand.

We used LARS for regularization partly because of its speed, partly because it scales well with increasing number of gene expressions, and partly because the least angle regression regularization technique fits directly into our linear model framework shown in (3). When multiple genes are involved in the regulation of metabolite production several of the corresponding gene expression profiles could have very similar expression patterns. In this case, even if all are influential then LARS will tend to select only one of the genes from this set at random while setting the coefficient of the remaining genes to zero. An alternative would be to use OSCAR (octagonal shrinkage and clustering algorithm for regression) which tries to address this issue by performing selection and clustering of correlated predictors simultaneously [15]. OSCAR assigns coefficients to all predictors in the model, which can then be ranked by the size of the assigned coefficients. Likewise the clustering is performed by assigning nearly identical coefficients to co-selected genes.

A disadvantage of our proposed method (as well as other variable selection methods) is that we consistently consider the first selected gene regardless of its strength or significance. Thus, we are guaranteed to find the “best association” from the available data but is unable to directly infer if an association appears to be stronger than what could be expected by chance. Our simulation results indicate that a variable importance approach based on resampling can be used to determine the strength of an association. One way to investigate the robustness of a result would be to run the analysis several times with different subsets of the genes, in the same way as with simulated data. If the same genes are selected irrespective of the subset they appear in, one would be more inclined to trust the effect of these genes to be “true” [16].

Multi-way models are not quite as numerous as shrinkage models, but alternatives to PARAFAC are available. Tucker models are often used to compensate for base shift in spectra [17]. However, we assume that the data has been sufficiently pre-processed for the metabolite spectra to comparable before analyzing the data so this is not a huge advantage. Non Negative Tensor Factorization (NTF) is a generalization of Non Negative Matrix Factorization aimed at retrieving underlying components from high dimensional data [18]. The basic formulation NTF is a PARAFAC model with non-negativity restrictions, while the extensions NTF1 and NTF2 produce different dimensional output, most importantly one matrix and one tensor, while PARAFAC produces three matrices [19]. The mixing matrix can still be extracted for the NTF1 and NTF2 extensions so NTF-based methods would all be potential alternatives within our framework. However, at this moment the computation time can be prohibitive so in practice we have to wait for some faster general purpose graphics processing unit implementations of NTF that are underway [20].

Although other studies attempt to model associations between genomic and metabolic data, none of these are directly comparable to what we present in this paper as they use data in different forms as well as incorporating prior knowledge [4], [6]–[8]. Methods based on PCA or PLS cannot be directly compared to our proposed method as the original data is reduced to components that are a mix of variables so it is impossible to infer a specific relationship between a single gene and a metabolite profile unless the principal components split up very advantageously [10], [11]. Unlike many other methods, the modeling framework proposed here does not make use of prior knowledge and have very few assumptions, which makes analysis of less studied organisms more approachable.

In conclusion, the method presented here successfully embraces the complex structures of modern high resolution analysis machinery. Our proposed model imposes few restrictions on the association between the two datasets and is more flexible than models based on correlation measures [7]. This allows for a more complex relationship between transcription and metabolite production. We also allow the model formulation to include more complex (*e.g.* interaction) terms without altering the basic concept. Each individual data type (spectra and gene expression) can be utilized to full extent and we can bridge the gap between transcriptomics and metabolomics in order to provide information as to which specific genes are involved in the underlying system pathways. This is a promising direction for large-scale analysis in the future, potentially eliminating separate modeling of different data types that essentially are part of the same system. The ability to choose different methods for dimension reduction and modeling as well as inclusion of treatment or other external variables makes this approach very flexible and applicable throughout the biological sciences.

## Methods

We wish to construct a statistical model that can model the observed metabolite data as a function of the observed gene expression data.

Apart from measuring two aspects of the same system, the two methods also produce different sized output, which is briefly described below. The metabolite data are assumed to arise from an LC-MS experiment, where the liquid chromatography separates the chemical compounds and measures elution time while the mass spectroscopy fragments the eluded compounds into smaller molecules summarized by their mass/charge ratio, 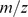. For each combination of elution time and 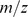 value, an intensity representing the amount of a given metabolite is measured. Thus, for a single experimental unit the resulting metabolome data can be represented as a matrix where each column represents a specific elution time, each row a particular 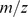 value, and the values in the data matrix are the observed intensities. The metabolome data from a single experimental unit can therefore be visualized as a three-dimensional plane with intensities giving the 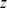 values. An example of such a plane is shown in Figure S1. Several samples will result in a three-dimensional tensor 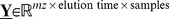 that will be part of our outcome/response data.

Gene expression studies measure the expression of thousands of genes simultaneously. Microarray experiments, for example, emit light proportional to the amount of RNA bound at a specific probe when excited by a laser, and the resulting image is converted to numeric values for expression. The data structure from such an experiment for a single experimental unit is a vector of expression values – one for each gene/probe. For several experimental units in a sample the full expression data can be combined into a data matrix where each row represents a gene/probe and where each column represents an experimental unit. The values in the gene expression matrix is then the observed expression levels, and each row will therefore act as a possible predictor in our scenario.

Let 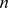 be the number of experimental units and assume that for each experimental unit data is obtained on *both* metabolic spectra as well as on gene expression levels. The three-dimensional tensor 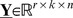 represents the values from the observed metabolic spectra from 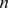 experiments measured at 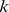 time points and at 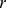 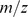 values and the two-dimensional tensor 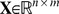 represents the corresponding observed gene expression values from 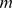 genes based on the 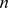 experiments. Note the 1:1 correspondence between the number and order of the units from the two datasets: 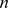 must be identical for both the metabolic spectra and the gene expression data for the approach to be meaningful.

We wish to model the expected metabolic spectra as a function of the gene expression levels in order to determine which genes are influencing the spectra, *i.e.*, such that
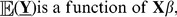
(1)where 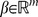 is a set of parameters describing the effect of each of the individual genes. If we can identify a set of genes with large coefficients then that set has an impact on the underlying biological system that produces the metabolic spectra.

The formulation in (1) poses two major problems: First, the dimension of the matrices comprising 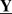 is typically extremely large compared to the number of experimental units, 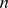, and at best we can only hope to identify and model the primary trends observed in 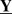. Consequently, we need to extract the primary components that constitute the observed spectra and model those as a function of the observed gene expression levels. Secondly, classical multiple regression techniques will fall short even if we are able to extract a set of spectra components to use as outcomes since the number of genes, 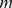, is also very large relative to the number of experiments, 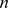. Hence, we need to apply dimension reduction techniques on both 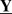 and 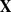 to extract the important aspects of the two types of data and to be able to model the relationship between the spectra, 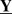, and the gene expression profiles, 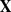.

Here, we suggest to decompose the spectra data, 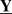, using PARAFAC to produce a three-way decomposition with 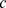 components [21], [22]. The number of desired components, 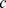, is chosen by the investigator and the decomposition produces matrices, 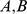, and 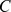 such that 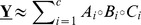, where 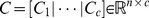 is called the mixing matrix, and where 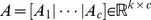 and 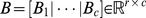 can be interpreted as basis functions for retention time and 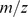 values, respectively. 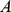 and 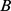 can be seen as the underlying building blocks of the mass spectrometry data in the sense that each column of 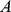 and 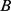 represents a building block profile, while 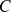 provides the amounts of each building block that is necessary to reconstruct 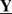.

The available gene expression values act as possible predictors in model (1), but since the number of genes, 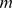, typically is orders of magnitude larger than the number of observations, 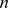, we need to make some kind of regularization on the regression parameters in order to prevent overfitting and to account for possible collinearity so we are able to identify a subset of genes of interest. Here we suggest to use least angle regression (LARS) as a way to apply constraints on the coefficient matrix of the model [23]. Least angle regression has the advantage that it is computationally very fast and that two predictors obtain coefficients that behave in the same way when they are equally correlated with the response. Thus, we can use LARS to select a small subset of genes from thousands of gene expressions and identify how they influence the mixing components of the spectra.

Our modeling idea is to employ two dimension reduction techniques: One to decompose the spectra into components that include basis functions and a mixing matrix and one that imposes restrictions on the vector of parameters 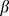 such that all parameters do not enter the model at the same time. Consequently, we can rephrase our model (1) as a set of 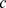 regression models (one for each of the mixing components):
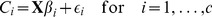
(2)

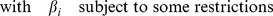
where we essentially model the mixing or weight of the basis functions as a function of the observed gene expression levels. Each 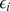 vector is assumed to follow an independent Gaussian distribution with mean zero and variance 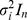. The restrictions applied to 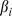 is due to prevent overfitting as defined by the least angle regression [23].

**Figure 4:**
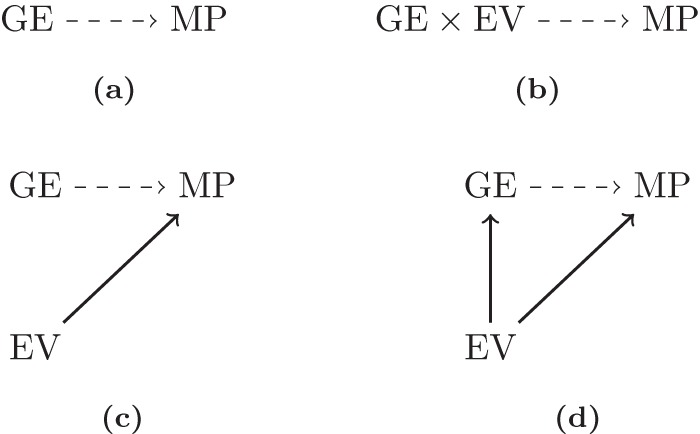
Relevant models when integrating gene expression and metabolic profile data. “GE”, “MP” and “EV” correspond to gene expression data, metabolic profile data, and external variables, respectively. The dashed lines represent the primary focus of interest: the association between gene expression levels and metabolic profiles while the solid lines represent possible associations between the available data.



Our primary interest is to identify any associations between the gene expression data and the metabolic profiles data so model (2) can be illustrated as shown in Figure 4a. Model (2) is extremely flexible, however, and we can easily include additional predictors based on the experimental design. If, for example, some of the experimental units were assigned to special treatment groups or belonged to specific genetic strains then we would like to model the effect of these external covariates. Two possible situations exists: either the external variables influence both the gene expression levels and the metabolic profiles (corresponding to the diagram shown in Figure 4d) or the variables may influence only the metabolic profiles (see Figure 4c). The former situation is often more realistic and corresponds to the situation where the external variables act as confounders. In either case, we can easily include external variables pertaining to the experimental design in our model when modeling each of the 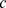 components in order to control for them by adding them to the model:

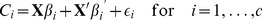
(3)

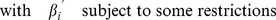


Here, the extra design matrix 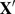 with corresponding parameters 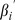 represents the external variables. Finally, the model also accommodates situations where external variables act as effect modificators of the gene expression levels (*i.e.*, the external variables change the effect of the gene expression data, see Figure 4b). That situation is handled by including an interaction between the gene expression data and the external variable in model (3). If the primary hypothesis of interest is how the external variables influence the metabolite profiles then the interaction can be used to answer that as well.

To summarize, our proposed approach consists of two stages:

1. Decompose the 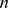 spectra into 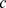 basis functions for retention time, 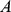, and 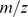 values, 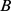, and the mixing matrix 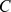.
2. Fit each of the 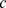 estimated mixing components as function of the observed gene expression values using a multiple linear regression model approach that accommodate overfitting (*i.e.*, can handle more regressors than observations).

In this paper we use parallel factors analysis (PARAFAC) to provide the three-way decomposition of the 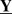 tensor while least angle regression (LARS) will be used for shrinkage regression and subset selection on the set of 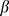 parameters from 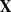. Other methods, like singular value decomposition, or non-negative tensor factorization for the metabolome data decomposition and ridge regression, or OSCAR (octagonal shrinkage and clustering algorithm for regression) for the shrinkage of the gene expression data could essentially be applied as well depending on a given situation. We consider alternative choices further in the discussion.

If an association between a gene expression level and a mixing component (*i.e.*, a 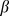 coefficient is found to be non-zero for one of the 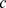 regression models) then that suggests that the corresponding gene is associated with the metabolites corresponding to the peaks in the relevant basis functions. For example, if we analyze, say, mixing component 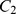 and find that 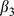 is non-zero for that component then this suggests that gene 3 influences the weight of basis functions 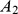 and 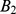. Basis function 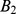 consists of a full metabolite profile so gene 3 will be associated to the full metabolite profile, but since the matrix decomposition separates the profiles based on the peaks in the spectra we essentially have that gene 3 is associated to the peaks (and the corresponding metabolites they represent for a given 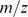 value) found on basis function 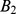. The same is true for basis function 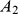. An important feature of the tensor (spectra) decomposition is that the two basis functions 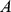 and 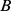 retain the 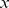-values of the original spectra. Subsequently, a table of compounds corresponding to the peaks found in the basis functions can be produced and metabolite databases such as Metlin can then be searched for compounds with these attributes. Thus, the individual component characteristics are intact and both chemical compounds of interest and the genes controlling their production can be identified from the model.

### Simulations

A simulation study was undertaken to demonstrate the validity of the proposed method, and data were generated as described below. Background and treatment effects were all arbitrarily chosen to create a data structure resembling what might be observed with real data, but the numbers have no further interpretation. Artificial treatments were constructed to make the gene expression data resemble what could be observed by using different experimental conditions. We assume that measurement error such as variation between microarrays, retention time shifts can be remedied by pre-processing and do not impose these on the simulated data.

The simulation procedure was as follows:

1.

(a) Gene expression data resembling output from a DNA microarray experiment were generated first. In this case we used 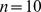 biological replicates and 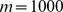 genes to represent the results from a small DNA microarray chip. The average expression level for each gene was drawn from a Gaussian distribution with mean 500 and standard deviation 4, 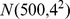. These 1000 simulated gene levels background were repeated 10 times to form the replicates.

(b) To simulate three groups of genes: up-, down-, and unregulated, we applied two different background levels (+225 and −180), to 400 and 500 randomly selected genes (rows) respectively. Secondly, two treatments (+100 and −320) were applied to 5 and 4 randomly selected replicates (columns) respectively. Finally Gaussian noise with standard deviation 100 was added to all observations, and absolute values used. Note that the up-, and down-regulated genes have an effect of approximately 2 standard deviations.

2.

(a) 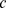 basis spectra 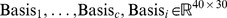, each containing one peak were generated by first simulating random Gaussian 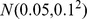 background noise. The coordinates of the peaks were found by randomly drawing 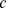 positions and they were sorted to make subsequent matching simpler. At each position a Gaussian 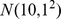 density replaced the original values to create a building block peak.

The dimension was arbitrarily chosen but kept small to make computations fast, and 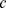 was varied in the following from 5 to 15 to represent different complexities in the spectra. We chose to let one peak represent the rows normally seen i LC-MS as these can be assume to arise from the same compound.

(b) Each basis spectra was scaled by the first 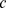 genes, *e.g.*, in the case of 5 basis spectra, the expression levels of each of the first 5 genes would influence the height of each of the 5 peaks in the “observed” spectra, thus, the observed spectra for sample 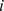 is given by
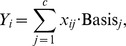
where 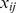 is the gene expression level from gene 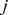 from sample 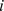. An example of a simulated spectra is shown in Figure 3. The scaled basis spectra, 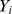, were combined to make a 3-D tensor 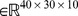


After simulation of the gene expression and metabolic data we applied the proposed method to see if we could identify which genes that are associated with each of the peaks in the metabolic spectra.

1. The simulated spectra were separated into basis and mixing matrices with a PARAFAC model using 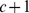 components. The extra component is meant to capture the background noise of the spectra. In practice, the true number of components, 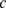, is not known but the number of peaks can be visually determined from the spectra, so it is not an unreasonable assumption that 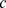 is known.
2. Seven different noise levels were applied to the simulated DNA microarray data to represent measurement error from the gene expression experiment. At each noise level, Gaussian noise with a standard deviation ranging from 0 to 60 in steps of 10 were added to 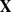 prior to using the data for gene identification in the LARS model (but after the true gene expression level had been used to influence the peak height).
3. Finally, the mixing matrix was used as response in 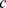 LARS regression models where the “noise component” from the decomposition is disregarded.

The first selected gene was compared to the true gene for each LARS model. PARAFAC does not retain the order of the peaks, thus the components of the mixing matrix were sorted according to the corresponding peak order of the basis matrix, 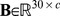. This ordering is purely to ensure that we can verify if the correct gene is matched with the correct peak as per step 2 (c) above.

This setup allows for rapid calculations and the LARS model produces compact output which is ideal since only the first gene selected by each of the LARS models is of interest in this study (*i.e.*, for the 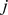
'th component we hope to find 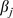 as the first selected gene). Explained variation in the PARAFAC decomposition was very high in all cases, 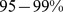. We quantify the noise added to the gene expression using (4), where 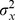 is the variance of the simulated gene expressions, 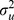 is the variance of the added noise, 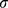 is the residual error from the LARS model and 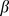 is the matrix of coefficients from the LARS model.
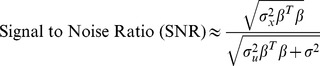
(4)


Our initial parameters for the simulation results in 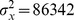, 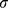 was found to be between 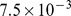 and 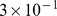 and 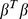 was between 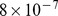 and 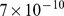. The calculated SNR values are found on the 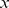-axis of Figure 1. A SNR value of 1 corresponds to equal variation in gene expression and background.

### Application

We applied the method to a Cassava (*Manihot esculenta*) dataset with 32 samples of leaf material of both LC-MS and single-color microarray data. Cassava is an important tuber crop in Africa, Asia and South America but due to its content of the toxic cyanogenic glucosides extensive processing is required before consumption. In spite of its socio-economic importance and identification of some of the processes in the catalyzation of cyanogenic glucosides [24] many pathway steps are yet unidentified. Likewise, gene annotation is far from complete; Roughly 3% of the genes in Phytozome (May 2013) are labeled ‘unknown protein’, and 15% have no annotation. Thus identification of genes associated with the compounds of interest could target potential areas of interest, which might eventually lead to non-toxic varieties.

Of the 32 samples, two samples were disregarded due to insufficient quality. A control sample revealed the region of interest in the LC-MS spectra, an example of which is shown in Figure S1. The 30 individual spectra shoved little variation with regard to peak height. After cropping and combining the spectra, the resulting dimension was 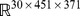. Gene expression of 13865 genes of which 30% were not annotated were measured in the microarray experiment for each of the 30 samples. The expression data was used without any preprocessing, such as normalization or alignment.

A three component PARAFAC model explained 90.71% of the variation in the LC-MS spectra. This was expected as 3 major peaks can be visually identified in the spectra (see Figure S1). Following our approach from the simulation study, a four component model would have been ideal, however the signals in the spectra was much stronger than in the simulated data. Thus, an additional component would not capture the noise but be a replicate of one of the other three components. Each column of the 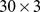 mixing matrix was subsequently used as response in 3 LARS models with all 13865 genes as potential predictors.

The first peak in the spectra was identified as Linamarin, a well-known compound in Cassava. The LARS regression selected a gene involved coupled to several CYP79 enzymes, which has been identified as a catalyst in the synthesis of Linamarin in Cassava [24]. With further study we hope to identify one of the other peaks as Lotaustralin and using the selected genes to further elucidate its synthesis pathway.

## Supporting Information

Figure S1 **LC-MS data, one biological sample.** Each\row” of peaks, illustrated by the red line, corresponds to a molecule fragmented into smaller parts. The width of the red line is exaggerated, as a single time point would be to narrow to be seen. Often one such row consists of many small peaks, and are analyzed one at a time.
(EPS)
